# The methods and baseline characteristics of a multi-site randomized controlled trial evaluating mindfulness-based relapse prevention in conjunction with peer support to improve adherence to medications for opioid use disorders

**DOI:** 10.3389/fpsyt.2024.1330672

**Published:** 2024-06-21

**Authors:** Mercy Ngosa Mumba, George Tongi Mugoya, Rebecca S. Allen, Andrea L. Glenn, Joshua Richman, Anchal Ghera, Austin Butler, Blossom Rogers, Teresa Ann Granger, Lori L. Davis

**Affiliations:** ^1^ Capstone College of Nursing, University of Alabama, Tuscaloosa, AL, United States; ^2^ Tuscaloosa VA Medical Center, Tuscaloosa, AL, United States; ^3^ Department of Educational Studies in Psychology, Research Methodology, and Counseling, University of Alabama, Tuscaloosa, AL, United States; ^4^ Department of Psychology, University of Alabama, Tuscaloosa, AL, United States; ^5^ Department of Surgery, University of Alabama at Birmingham Heersink School of Medicine, Birmingham, AL, United States; ^6^ Birmingham VA Health Care System, Birmingham, AL, United States; ^7^ Department of Psychiatry, University of Alabama at Birmingham Heersink School of Medicine, Birmingham, AL, United States

**Keywords:** opioid use disorder, mindfulness based relapse prevention, MOUD, buprenorphine, relapse, substance use disorder, twelve-step facilitation

## Abstract

**Introduction:**

Medications for opioid use disorders (MOUD) remain the gold standard for treating OUD, but treatment initiation and adherence remain challenging. Exclusive utilization of pharmacotherapy as a treatment modality for OUD is sub-optimal, and a combination of psychotherapies and pharmacotherapies is recommended. General trends indicate the benefits of peer mentoring and MBRP separately. Therefore, we hypothesize that the combined effect of MBRP and Peer mentoring will produce synergistic improvements in MOUD adherence compared to an enhanced twelve-step facilitation (TSF).

**Methods:**

This paper describes the methods and baseline characteristics of a multi-site randomized controlled trial evaluating the effectiveness of a combination of MBRP and peer support (MiMP) compared to an enhanced TSF in improving adherence to MOUD. Both MiMP and TSF are 12-week manualized protocols that utilize licensed therapists. The interventions are delivered in weekly group sessions that last about 75–90 minutes per session. The primary outcome is MOUD adherence. Secondary and exploratory outcomes include relapse, cravings, depression, anxiety, stress, quality of life, and pain catastrophizing.

**Results:**

The participants’ ages ranged from 21 years to 77 years, with a mean age of 44.5 (SD ± 11.5 years). There was an almost equal distribution of gender and place of residence. Overall, 51.9% (n=54) of participants identified as female and 48.1% (n=50) were male. Similarly, 51.9% (n=54) of participants resided in urban areas, while 48.1% (n=50) resided in rural areas. Participants identified as either black or white, with over three-quarters identifying as white (77.9%, n= 81) and 22.1% (n= 23) as black. Most participants randomized to the 12-step facilitation group were white (93.1%). Relationships and employment status were well distributed between categories. Over half of the participants reported some college or higher education. Over 90% of the participants made less than $75,000 per year. Some participants indicated that they had both public and private health insurance.

**Discussion and conclusion:**

This study is innovative in several ways including combining MBRP and peer support, addressing comorbid mental health issues among individuals with OUD, utilizing manualized protocols, and evaluating of both physiological and self-reported measures in assessing cortisol reactivity as a predictor of relapse and treatment outcomes.

## Introduction

For three years in a row, drug overdose deaths in the United States have exceeded 100,000 and almost 70% of those deaths are related to fentanyl and other emerging drugs such as psychostimulants and xylazine (1–3). Alabama is one of the few states where drug overdose deaths continue to rise in the United States, representing a nearly three-fold increase from the previous year (4). These estimates are even more pronounced in rural areas where the negative impacts of social and structural determinants of recovery are endemic and continue to produce poor outcomes for rural populations with SUD. Although the drug use landscape in the Unites States is ever evolving, opioids still account for about 70% of drug overdoses (NIDA, 2023). Unfortunately, Alabama still has one of the highest opioid dispensing rates in the nation, with some counties reporting dispensing rates as high as 139 prescriptions per 100 persons. (5).

Medications for opioid use disorders (MOUD) remain the gold standard for treating OUD, but treatment initiation and adherence remain challenges for researchers, practitioners and clinicians alike. Medications such as buprenorphine (various formularies), methadone, and naltrexone are some of the most commonly used to treat OUD. Behavioral interventions have also shown promise in improving treatment outcomes for individuals with SUD. For example, many studies have explored behavioral interventions including cognitive behavioral therapy (CBT; 6), motivational interviewing (MI; 7), and contingency management (CM; 8) in the treatment of SUD. Recently, some studies have explored the utility of mindfulness-based relapse prevention (MBRP) in the treatment of behavioral health and substance use disorders (9–13).

Given research findings indicating that exclusive utilization of pharmacotherapy as a treatment modality for OUD is sub-optimal, a combination of psychotherapies and pharmacotherapies is recommended (14, 15). Preliminary evidence suggests that mindfulness could improve substance use treatment outcomes. However, several limitations have been identified in more recent studies that combine pharmacotherapy and psychotherapy which have found non-significant results, including (a) provision of counseling by individuals without addiction expertise (b) providing counseling based on manuals that address addictions in general or that are outdated, and (c) outcomes that have exclusively relied on self-report measures. The present study addresses these identified limitations.

## Materials and methods

### Overview of design, aims, and hypotheses

This is a multi-site randomized controlled trial evaluating the effectiveness of a combination of MBRP and peer support (MiMP) compared to an enhanced TSF in improving adherence to MOUD. The study is approved by the institutional review board (IRB) at the University of Alabama, the Tuscaloosa VA Medical Center, and the Birmingham VA Medical Center. All participants provide informed consent prior to participating in any study procedures. The study has two main objectives: 1) determine whether the MiMP improves adherence to MOUD (primary outcome) and reduces relapse, cravings, self-reported stress, depression, anxiety (secondary outcomes measures), and reduces cortisol levels and cortisol reactivity to drug cues (exploratory outcome measures), and 2) examine whether pre-intervention cortisol reactivity is predictive of relapse outcomes, and/or if reductions in cortisol reactivity over the course of intervention mediate relapse outcomes. The central hypothesis for this study is that individuals receiving the MiMP will show greater adherence to MOUD (Ha), reduced rates of relapse (Hb), cravings (Hc), as well as improvements in other secondary and exploratory outcome outcomes.

### Eligibility criteria

The MINI International Neuropsychiatric Interview for DSM-5 (MINI-5; 16) is an updated semi-structured clinician-administered interview for DSM-5 diagnoses that is conducted at baseline to confirm eligibility criteria for OUD and document comorbid diagnoses. During the screening phase, potential participants are also screened for other inclusion and exclusion criteria. To be included in the study individuals must be at least 19 years old, have a diagnosis of OUD, and must be currently receiving MOUD (all types of MOUD are accepted) from an established provider. Participants may meet criteria for mood, anxiety, or other psychiatric disorders based on the DSM-V criteria and are able to participate if they are clinically stable. This is because a significant number of individuals with SUD present with comorbid mental and behavioral health conditions. Participants must also be able to read and understand English as all our study procedures are conducted in English.

Exclusions criteria for the study include significant cognitive impairment, actively suicidal or homicidal, active psychosis, and/or unstable medical conditions that contraindicate proposed treatment. Subject exit criteria include increases in alcohol or drug use leading to the need for a more intensive level of care (i.e., medical detoxification or inpatient treatment), inability to manage psychiatric symptoms within the inclusion/exclusion criteria of the study (i.e., need for the initiation of maintenance psychotropic medications; development of psychosis), and inability to return for therapy sessions due to incarceration or hospitalization.

### Study intervention arms

#### The Minds and Mentors Program

The current study tests a theoretically and empirically informed novel intervention. It focuses on the integration of care models among treatment facilities, clinicians, community-based support services, peer mentors, and patients, thereby creating a supported ecosystem of treating OUD in Alabama. The MiMP intervention is informed by (a) previous studies, (b) guidelines from SAMHSA and NIDA, (c) input from experts in the field (both researchers and clinicians), and (d) addresses the pitfalls identified in the current literature. Further, this treatment is unique because, in addition to addressing OUD, it addresses many comorbid mental and behavioral health problems that have been found to complicate treatment outcomes.

The MiMP intervention is a combination therapy of therapist led MBRP and peer mentor facilitation among individuals with OUD who are currently on MOUD. The intervention utilizes a 12-week manualized MBRP protocol, while incorporating cognitive-behavioral skills (i.e., effective coping skills, self-efficacy, and recognizing common antecedents of relapse) with mindfulness-based practices to decrease the probability of relapse by increasing awareness and flexible responding in the presence of substance use triggers. The first eight (8) weekly sessions are offered consecutively and are led by a licensed therapist with experience working with individuals with substance use (preferably opioid use) in collaboration with a peer mentor. Both the licensed therapists and the peer mentor are trained by a study co-investigator who is an expert in MBRP, and this investigator conducts all fidelity monitoring for MiMP treatment arm. Following the completion of the first eight (8) weeks of the MBRP sessions (conducted by the therapist and per mentor), participants attend four (4) peer led sessions (offered every week for four weeks without a therapist). The purpose of the peer led sessions is to begin transitioning study participants to community-based resources and to established therapeutic relationships with the peer mentor who continues to support the participants by checking on them on a weekly basis after the end of the active intervention phase. Each MBRP and peer led session lasts for approximately 75–90 minutes. Peer support specialists are required to be certified by the state, has at least five years of sobriety, experience working directly with individuals in recovery, possess group facilitation skills, and are willing to be trained in mindfulness-based relapse prevention. There is only one peer support specialist and one licensed therapist per group.

#### Theoretical framework for the MiMP

The hallmark of mindfulness-based relapse prevention (MBRP) is the intentional attending to experiences in a non-judgmental manner that promotes non-reactive states of awareness (9, 17). Advantages of MBRP over usual relapse prevention (RP) interventions include the emphasis on approach-based goals, controlling of negative affect and craving, and recognizing underlying reasons for maladaptive behaviors (9, 17). In a randomized trial comparing MBRP to usual continuing care in 168 individuals who had completed acute care treatment for SUDs (18), the MBRP group reported significantly fewer days of drug or alcohol use (2.1 versus 5.4 days) and significant reductions in craving in the two months following the intervention. Additionally, reductions in cravings partially mediated decreased substance use and the MBRP group was less likely to crave in response to depressed mood (19), but the MBRP impact was not explained by improvements in depression.

Despite all the positive outcomes associated with MBRP, several limitations have been identified in previous research. In a meta-analysis of mindfulness-based treatments for addictive disorders conducted by Garland and Howard (20), although preliminary evidence was largely positive, many of the studies evaluated had serious limitations, including small sample sizes, high attrition rates, relied extensively on self-report measures for substance use and other constructs, and lack of post-treatment and follow-up interviews of personal experiences with the programs. Additionally, conclusions about efficacy are difficult because of a lack of standardized outcomes for these studies and heterogeneity of interventions. Recommendations to counteract these limitations include further studies using high-quality research methods including a written treatment manual.

**Figure 1:**
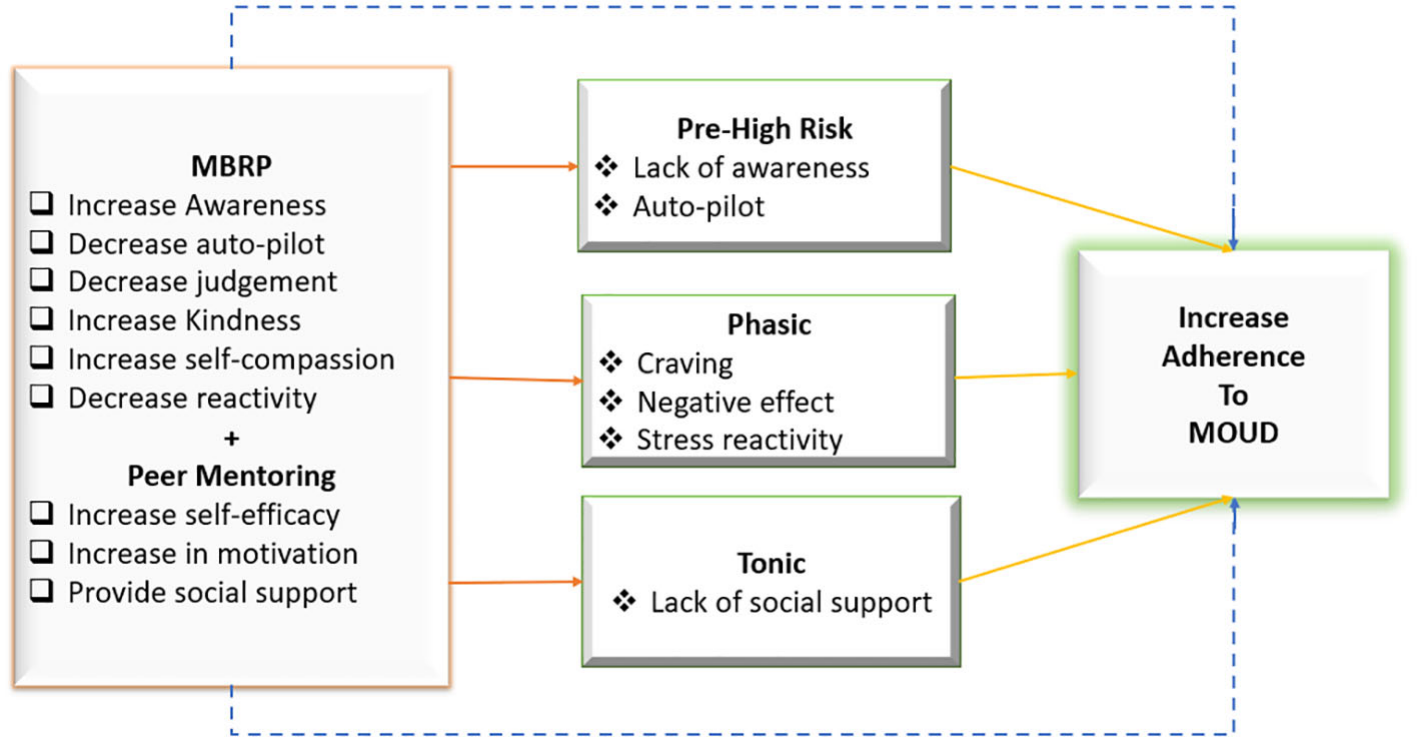
Conceptual framework for the minds and mentors program.

Adherence to MOUD and SUD treatment in general is often problematic and leads to poor outcomes in this population. To combat this problem, peer mentoring for SUD is gaining considerable support. Peer mentoring for substance abuse has also been recognized by the Substance Abuse and Mental Health Services Administration (21) under their Recovery Community Services Program and the Access to Recovery initiative (ATR) as improving treatment outcomes for SUD. Advantages of peer support in SUD treatment include improving connectivity to outpatient services for individuals with SUD, decrease in dependence on clinical staff for aftercare and follow-up, improved accountability for patients, increase in post-discharge treatment attendance, and facilitation of the formation of empathetic and therapeutic relationships (21, 22). Given the general trends indicating benefits of peer mentoring and MBRP separately, we hypothesize that the combined effect of MBRP and Peer mentoring will produce synergistic improvements in MOUD adherence compared to an enhanced twelve-step facilitation (TSF) and will lead to improvement in other psychosocial indices such as cravings, depression, anxiety, and stress (**Figure 1**). We call this combined intervention the Minds and Mentors Program (MiMP).

This theoretical model (**Figure 1**) extends beyond Witkiewitz and colleagues (2014) explanations of relapse prevention and thus informing how mindfulness increases adherence to MOUD by positing that in addition to offering participants MBRP, peer mentoring is an important component to preventing and/or reducing substance use. Thus, in addition to the phasic processes, which are the immediate precipitants of relapse within a high-risk situation that increase a person’s vulnerability in the moment, we hypothesize that MiMP may be able to target the tonic processes, which encompass various risk factors that represent underlying vulnerability for resumption of substance use and misuse. This represents social support in our study. We hypothesize that MBRP will directly prevent occurrence of a high-risk situation and directly reduce phasic risk (solid lines from MBRP), which will in turn lead to a decrease in substance use and adherence to MOUD. Additionally, MBRP will directly prevent substance use/relapse (dotted line). Secondly, peer mentoring will directly prevent tonic risk by specifically improving social support (solid line from peer mentoring), which will in turn lead to a decrease in substance use. Additionally, peer mentoring will directly prevent substance use/relapse (dotted line). We will test these hypotheses and mechanisms as part of our overall analytic plan upon study conclusion.

### Twelve-step facilitation

The comparison group consists of participants attending an enhanced TSF. TSF is modeled on the 12-step program, which involves helping participants with understanding and incorporating the core principles of 12-Step approaches into their recovery process while at the same time encouraging active participation in 12-Step meetings and related activities. The TSF is enhanced via the incorporation of CBT and MI tenets to ensure equipoise between the treatment arms. Compared to treatment as usual (TAU), TSF has been shown to significantly improve abstinence in substance use treatment (OR 2.44, p<0.05; 23). Additionally, TSF promotes abstinence by facilitating the patient’s acceptance and surrender of substance misuse as well as other addictive behaviors. The TSF used in this research effort is based on a manual that was originally developed for individual sessions and has since been adapted for group delivery by Brown et al. (24). Like MiMP, TSF involves 12 weekly sessions that are facilitated by a licensed counselor. There is no peer support in the TSF treatment arm. The TSF interventionists are trained by a different study co-investigator, who also completes all fidelity monitoring procedures for this treatment arm. Each TSF session lasts for approximately 75–90 minutes.

### Randomization and blinding procedures

To minimize the lag between recruitment and beginning the group-based intervention, randomization was done in units of five consecutive enrollees. Permuted block randomization stratified by recruitment site was used to assign each unit with block sizes of 2 and 4. The randomization assignment for each 'unit' of five consecutive participants was revealed only after each unit (group of 5) had enrolled. The study PI and biostatistician are the only research members who know the randomization schedule. Therefore, all research staff completing screening and baseline assessment for study participants are blinded to the randomization. Study participants are only randomized when they have completed all screening and baseline assessment, and a group of five participants are ready to start group interventions. Therefore, there is concealment of allocation of study participants all the way until study participants are ready to begin the intervention phase. The study interventionists are not blinded.

### Fidelity monitoring

Therapists and peer mentors receive supervision in-person or via teleconference. Each interventionist is monitored by the corresponding investigator with associated expertise in the treatment modality. These check-ins focus on treatment model adherence, quality of the intervention being delivered, barriers and facilitators of the group sessions, and any clinical concerns about participants. Therapist and peer mentors are provided with resources and ideas on reducing departure from the treatment protocol. Prior to the check-ins, the therapists and peer mentors receive the applicable session rating forms, so they are clear on what aspects they are evaluated. The goal is to achieve acceptable rating scores. Each treatment arm has a validated checklist that is utilized (25, 26). Additional training sessions are scheduled if it is determined that the therapist or peer mentor is not adhering to the treatment manual and meeting other fidelity measures. Every attempt is made to correct problems through training and supervision.

### Clinical assessments, outcome measures, and anticipated analyses

The primary outcome variable is adherence to MOUD, quantified as the number of days MOUD was received as indicated during the last 4 weeks of the intervention period. We expect that this can be treated as a continuous variable in linear regression models. However, prior to modeling we will examine its distribution, to determine if this is appropriate. If, for example, we observe large ceiling effects (many participants report very high or perfect adherence) then we may model non-adherence as the number of days when MOUD was not received. In this case we expect it would have something similar to a Poisson distribution, possibly with over-dispersion which would suggest the use of negative binomial regression.

The secondary outcome is relapse, which will be treated in two ways (a) a binary indicator if any relapse during the last 4 weeks of the intervention period is reported as determined by UDS or self-report, and (b) the total number of self-reported days of use during the last four weeks of the intervention period. The first dichotomous outcome will require logistic regression. The second approach will depend on the observed distribution and may be treated as either negative binomial or continuous. Other secondary and exploratory outcome variables include depression, anxiety, perceived stress, community integration, quality of life, sleep, pain severity, pain intensity, pain interference, and pain catastrophizing (**Table 1**).

**Table 1:** Study secondary and exploratory outcomes.

Instrument	Construct	Instrument	Construct
Opioid Craving Scale	Craving	PROMIS Pain Intensity (Scale 0-10)	Pain Intensity
Perceived Stress Scale	Stress	PROMIS Pain Interference	Pain Interference
Generalized Anxiety Disorder 7	Anxiety	PROMIS Pain Catastrophizing	Pain Catastrophizing
Patient Health Questionnaire-9	Depression	Sleep Scale from the Medical Outcomes Study	Sleep Problems
PROMIS Quality of Life	Quality of life		

We also collect saliva samples as a proxy for cortisol reactivity to examine whether baseline cortisol predicts relapse at the end of the study. Two samples are collected at each of the five-time points for data collection. At each time point, a participant provides a saliva sample at baseline and after watching a 20-minute video depicting drug use and other paraphernalia to examine cortisol reactivity. Findings of the cortisol testing will be included in a different paper since cortisol is utilized to examine its predictive capacity for relapse at the end of the study. Therefore, the discussion in this paper is limited to the novelty of having both physiological and self-report measures for cortisol in our study protocol.

We collect data from participants at five different time points. The data collection timeframes are as follows: T0: Baseline, T1: Week 8 of intervention phase, T2: Week 12 (end of intervention), T3: 3-month follow-up (12 weeks post intervention), and T4: 6-month follow-up (24 weeks post intervention). For this paper, we only report data for T0 – baseline characteristics of study participants to date since the study recruitment is ongoing.

Upon study completion, we will assess missing data and employ an intent-to-treat analysis. The planned analysis using random effects will overcome some of the limitations due to missing data. However, if missingness is problematic and plausibly missing-at random, analyses will be repeated using multiple imputation by chained equations as a sensitivity analysis. Prior to the formal analysis of outcomes, all data will be examined using statistical graphs and summary statistics (e.g., mean, standard deviation, median, etc.) and statistical graphs.

### Sample size calculations

This study is powered on the primary hypothesis that there will be a clinically significant increase in adherence to MOUD quantified by the number of days the participant reports adherence to MOUD in the last four weeks of the intervention period. Secondary outcomes which we expect to be similarly powered are decrease in relapse in the same period (treated dichotomously as any vs. none, and as a count of the number of days), and reduction in cravings in individuals receiving the MiMP compared to the TSF. Repeated measures for each person should also result in increased power.

The study is powered to detect the least of the effect sizes determined to be clinically meaningful for the study hypotheses, which we determined would be a medium effect size (d = 0.5). In a study of mindfulness-based relapse prevention for substance use disorders, Bowen et al. (18) found that 73% of the sample (N=168) returned for 4-month follow-up assessments. Additional studies have found retention rates over 80% at 3-month follow-up for participants receiving MOUD undergoing behavioral interventions (27). In the proposed study we expect an attrition rate of 20%. Therefore, we should achieve adequate power to detect clinically meaningful risk reductions at the pre-specified time points by randomizing 120 subjects to each arm of the study (N=240 total subjects).

## Results

**Table 2** highlights the socio-demographic characteristics of the participants. The participants’ age ranged from 21 years to 77 years, with a mean age of 44.5 (SD ± 11.5 years). There was an almost equal distribution of gender and place of residence. Overall, 51.9% (n=54) participants identified as female and 48.1% (n=50) male. Similarly, 51.9% (n=54) of participants resided in urban areas, while 48.1% (n=50) resided in rural areas. Participants identified as either black or white, with over three quarter identifying as white (77.9%, n= 81) and 22.1% (n= 23) black. It is noteworthy that a vast majority of participants randomized to the TSF group were white (93.1%). Relationships and employment status were well distributed between categories. Over half of the participants reported that they have some college or higher education. Over 90% of the participants made less than $75,000 per year. Some participants indicated that they had both public and private health insurance.

**Table 2:** Baseline sociodemographic characteristics.

	MiMP (*n=34*)		12-Step (*n=29*)		AR (*n=41*)		Total (*n=104*)	
	N	%	N	%	N	%	N	%
Participant Age								
Mean Age (SD)	46.9 (13.3)		43.6 (9.8)		41 (43.3)		44.5 (11.5)	
Age range	21 - 75		23 - 63		26 - 77		21 - 77	
Gender at Birth								
Female	17	50.0	17	58.6	20	48.8	54	51.9
Male	17	50.0	12	41.4	21	51.2	50	48.1
Ethnicity								
Black	10	29.4	2	6.9	11	26.8	23	22.1
White	24	70.6	27	93.1	30	73.2	81	77.9
Residence								
Rural	17	50.0	13	44.8	20	48.8	50	48.1
Urban	17	50.0	16	55.2	21	51.2	54	51.9
Relationship Status								
Divorced	13	38.2	6	20.7	9	22.0	28	26.9
Married	8	23.5	5	17.2	14	34.2	27	26.0
Never Married	6	17.7	7	24.1	10	24.4	23	22.1
Separated	5	14.7	6	20.7	4	9.8	15	14.4
Widowed	2	5.9	5	17.2	4	9.8	11	10.6
Education Level								
Less than high school	4	11.8	7	24.1	9	22.0	20	19.2
High school/GED	6	17.7	9	31.0	10	24.4	25	24.0
Some college	16	47.1	12	41.4	20	48.8	48	46.2
Bachelor's degree	5	14.7	1	3.5	2	4.9	8	7.7
Graduate/Professional	3	8.8	0	0.0	0	0.0	3	2.9
Employment status								
Employed	10	29.4	10	34.5	25	61.0	45	43.3
Unemployed	8	23.5	7	24.1	7	17.1	22	21.2
Disabled, unemployed	11	32.4	10	34.5	9	22.0	30	28.9
Retired	5	14.7	2	6.9	0	0.0	7	6.7
Income Level								
Less than $10,000	3	8.8	12	41.4	21	51.2	36	34.6
$10,000-$24,999	14	41.2	10	34.5	11	26.8	35	33.7
$25,00-$49,999	7	20.6	3	10.3	4	9.8	14	13.5
$50,000-$74,999	7	20.6	2	6.9	2	4.9	11	10.6
$75,000 and above	2	5.9	2	6.9	3	7.3	7	6.7
Prefer not to answer	1	2.9	0	0.0	0	0.0	1	1.0
Health Insurance								
Public Only	10	29.4	6	20.7	9	22.0	25	24.0
Private Only	16	47.1	13	44.8	12	29.3	41	39.4
Both Public & Private	1	2.9	8	27.6	9	22.0	18	17.3
No Insurance	7	20.6	2	6.9	11	26.8	20	19.2

AR, Awaiting Randomization to MiMP or 12-Step Facilitation.

**Table 1** highlights the socio-demographic characteristics of the participants. The participants age ranged from 21 years to 77 years, with an average mean age of 44.5 ((SD ± 11.5 years). There was an almost equal gender and place of residence. Overall, 51.9% (n=54) participants identified as female and 48.1% (n=50) male. Similarly, 51.9% (n=54) participants resided in urban areas, while 48.1% (n=50) resided in rural areas. Participants identified as either black or white, with over three quarter identifying as white (77.9%, n= 81) and 22.1% (n= 23) black. Its noteworthy that a vast majority of participants randomized to the 12-step facilitation group were white (93.1%).

Results for the utilization of MOUD are shown in **Table 3**. A vast majority of participants were using suboxone as MOUD, with over 72.8% of all participants reporting using suboxone. The next common MOUD used was methadone. There was an even distribution of length of time participants had been on MOUD. It is noteworthy that over a quarter of the participants had been using MOUD for over 2 years (49 months plus). **Table 4** contains results for baseline assessments. Most participant reported low craving at baseline with mean scores ranging from 1.19 (SD ± 1.94) among individuals awaiting randomization, 1.47 (SD ± 1.94) among individuals randomized to TSF, to 2.24 (SD ± 2.66) among participants randomized to the MiMP group. Both perceived stress and generalized anxiety mean scores were relatively evenly distributed among the various groups. Participants had high scores for quality of life, with individuals awaiting randomization having the highest reported quality of life mean score 23.05 (SD ± 2.66) and scores ranging from 13 to 26. Pain catastrophizing was relatively higher among the group randomized to MiMP (Mean score 10.26, SD ± 7.32) when compared to those in TSF (Mean score 7.55, SD ± 7.81) and awaiting randomization (Mean score 6.15, SD ± 7.57).

**Table 3:** MOUD use.

	MiMP (*n=34*)		12-Step (*n=29*)		AR (*n=41*)		Total (*n=104*)	
	N	%	N	%	N	%	N	%
Type of MOUD								
Buprenorphine	3	8.8	1	3.5	2	5.0	6	5.8
Methadone	2	5.9	6	20.7	7	17.5	15	14.6
Naltrexone	2	5.9	2	6.9	0	0.0	4	3.9
Sublocade	0	0.0	1	3.5	2	5.0	3	2.9
Suboxone	27	79.4	19	65.5	29	72.5	75	72.8
Length of time on MOUD in Months								
0.5—6	7	20.6	2	6.9	8	20.0	17	16.5
7—12	7	20.6	7	24.1	9	22.5	23	22.3
13—24	6	17.7	4	13.8	7	17.5	17	16.5
25—48	4	11.8	8	27.6	4	10.0	16	15.5
49 plus	10	29.4	8	27.6	12	30.0	30	29.1

AR, Awaiting Randomization to MiMP or 12-Step Facilitation.

A vast majority of participants were using suboxone as MOUD, with over 72.8% of all participants reporting using suboxone. The next common MOUD used was methadone. There was an even distribution of length of time participants had been on MOUD. Its noteworthy that over a quarter of the participants had been using MOUD for over 2 years (49 months plus).

**Table 4:** Baseline assessments of selected variables.

Variable	MiMP (*n=34*)				12-Step (*n=29*)				AR (*n=41*)			
	Mean	SD	Min	Max	Mean	SD	Min	Max	Mean	SD	Min	Max
Craving	2.24	2.66	0	8	1.47	1.94	0	5	1.19	2.49	0	10
Perceived Stress	18.74	7.79	1	35	19.28	8.86	0	35	18.63	7.04	6	35
Generalized Anxiety	9.62	6.46	0	21	9.55	6.53	0	21	8.85	5.93	0	21
Depressive symptoms	11.47	6.78	0	22	11.55	6.98	0	26	9.32	6.44	0	24
Sleep Problems	3.31	1.26	1	6	3.46	1.17	1	6	2.85	1.20	1	6
Pain Catastrophizing	10.26	7.32	0	24	7.55	7.81	0	24	6.15	7.57	0	24
Pain Interference	9.44	8.22	0	24	8.59	7.98	0	24	5.75	6.66	0	24
Pain Intensity	11.33	9.26	0	30	11.33	8.12	0	30	8.98	8.95	0	30
PROMIS-Quality of Life	21.35	5.26	9	26	20.90	5.01	11	26	23.05	4.45	13	26

AR, Awaiting Randomization to MiMP or 12-Step Facilitation.

Various assessments were conducted at baseline. Most participants reported low craving at baseline.

## Discussion

This study describes the methods and examines the baseline characteristics of a multi-site randomized controlled trial that compares the effectiveness of MiMP to TSF in improving adherence to MOUD, reducing relapse, as well as improving other psychosocial outcomes including depression, anxiety, stress, cravings, and pain catastrophizing, among others. We adapted the theoretical model for characterizing the mechanistic targets of mindfulness training (28) to develop the MiMP interventions and it will be utilized understand the mechanism through which the MiMP intervention may impact outcomes and eventually lead to an increase in MOUD adherence, upon study conclusion (See **Figure 1**).

Our study is innovative in many ways, especially how it targets limitations of previous studies. Utilizing licensed therapists who have expertise in treating individuals with OUD and comorbid mental and behavioral health problems is important. The added advantage of peer support provides a continuum of care that provides better transition to community-based resources, which is often a major criticism of randomized controlled trials whereby study participants feel abandoned after their participation in the intervention aspects of the study. Moreover, this transition offers better chances of widespread clinical adoption and translation of the intervention into real-world clinical settings, further contributing to closing the research-translation gaps that are inherent in OUD treatment settings.

Another innovation of the current study is that the intervention intentionally targets psychiatric comorbidity that commonly occurs with OUD, including depression, anxiety, and stress. By also targeting these comorbidities, this treatment has the potential to disrupt the link between stress, cravings, and relapse. For example, depression and anxiety increase the risk of OUD and have been found to double the risk of relapse among those undergoing treatment, indicating that individuals with these conditions require specialized attention while engaged in OUD treatment (29).

Functionally, stress-related comorbidities negatively affect OUD recovery by increasing cravings. Cravings are risk factors for both substance abuse initiation and relapse (30, 31). In fact, individuals who report sudden increase in cravings are approximately 14 times more likely to relapse than individuals who have a gradual increase in cravings (32). This emphasizes the need for treatments that target co-occurring mental health conditions for individuals with OUD. This is crucial because among people with OUD in the U.S., about 27% of them have a serious mental illness and another 64% report any mental illness diagnosis (NIDA, 2023). Despite recent increases in mental health services, barriers to accessing treatment persist (NIDA, 2023) especially in rural regions.

Our baseline demographic characteristics were somewhat different from other mindfulness based RCTs in the literature focused on substance use disorders (9, 18, 33). For example, our sample was predominantly white, had almost equal distribution between males and females, over half of the participants had some college education or higher, and only about a third of participants made less than $10,000 per year. In contrast, Bowen et al. (18) reported a more racially diverse sample including 15.3% of participants identifying as multicultural and 7.7% identifying as Native American. Bowen et al. (9) over 50% of their sample reported educational attainment of high school diploma/GED or lower, and higher rates of unemployment compared to our study participants. Lastly, Zemestani and Nikoo (33) reported lower mean ages compared to our study sample.

## Conclusion

The MIMP program is a theoretically informed and empirically supported novel intervention that promises to yield positive outcomes for individuals with OUD. The baseline characteristics of participants reveal similarities between groups which are important in examining intervention effects over time. This study is innovative in many ways and has the potential for widespread clinical adoption thereby reducing the research-translation gap of evidence-based interventions for treating OUD.

## Data availability statement

The raw data supporting the conclusions of this article will be made available by the authors, without undue reservation.

## Ethics statement

The studies involving humans were approved by The University of Alabama Institutional Review Board. The studies were conducted in accordance with the local legislation and institutional requirements. The participants provided their written informed consent to participate in this study.

## Author contributions

MM: Conceptualization, Data curation, Formal analysis, Funding acquisition, Investigation, Methodology, Project administration, Resources, Supervision, Validation, Visualization, Writing – original draft, Writing – review & editing. GM: Conceptualization, Data curation, Formal analysis, Funding acquisition, Investigation, Methodology, Project administration, Visualization, Writing – original draft, Writing – review & editing. RA: Funding acquisition, Writing – review & editing. AGl: Conceptualization, Data curation, Formal analysis, Funding acquisition, Investigation, Methodology, Writing – review & editing. JR: Conceptualization, Data curation, Funding acquisition, Methodology, Writing – review & editing. AGh: Investigation, Writing – review & editing. AB: Investigation, Writing – review & editing. BR: Investigation, Writing – review & editing. TG: Writing – review & editing. LD: Conceptualization, Data curation, Formal analysis, Funding acquisition, Investigation, Methodology, Project administration, Supervision, Visualization, Writing – original draft, Writing – review & editing.
